# Seminal vesicle secretory protein 7, PATE4, is not required for sperm function but for copulatory plug formation to ensure fecundity^†^

**DOI:** 10.1093/biolre/ioy247

**Published:** 2018-11-18

**Authors:** Taichi Noda, Yoshitaka Fujihara, Takafumi Matsumura, Seiya Oura, Sumire Kobayashi, Masahito Ikawa

**Affiliations:** 1 Research Institute for Microbial Diseases, Osaka University, Suita, Osaka, Japan; 2 Graduate School of Pharmaceutical Sciences, Osaka University, Suita, Osaka, Japan; 3 Institute of Medical Science, The University of Tokyo, Minato-ku, Tokyo, Japan

**Keywords:** artificial insemination, coagulating gland, mouse, semen leakage, sperm fertilizing ability, sequential mating

## Abstract

Seminal vesicle secretions (SVSs), together with spermatozoa, are ejaculated into the female reproductive tract. SVS7, also known as PATE4, is one of the major SVS proteins found in the seminal vesicle, copulatory plug, and uterine fluid after copulation. Here, we generated *Pate4* knockout (−/−) mice and examined the detailed function of PATE4 on male fecundity. The morphology and weight of *Pate4*−/− seminal vesicles were comparable to the control. Although *Pate4*−/− cauda epididymal spermatozoa have no overt defects during in vitro fertilization, *Pate4*−/− males were subfertile. We found that the copulatory plugs were smaller in the vagina of females mated with *Pate4*−/− males, leading to semen leakage and a decreased sperm count in the uterus. When the females mated with *Pate4*−/− males were immediately re-caged with *Pate4*+/+ males, the females had subsequent productive matings. When the cauda epididymal spermatozoa were injected into the uterus and plugged artificially [artificial insemination (AI)], *Pate4*−/− spermatozoa could efficiently fertilize eggs as compared to wild-type spermatozoa. We finally examined the effect of SVSs on AI, and observed no difference in fertilization rates between *Pate4*+/+ and *Pate4*−/− SVSs. In conclusion, PATE4 is a novel factor in forming the copulatory plug that inhibits sequential matings and maintains spermatozoa in the uterus to ensure male fecundity.

## Introduction

In mammals, spermatozoa generated in the testis cannot fertilize eggs until they acquire fertilizing abilities when they pass through the epididymis. Whereas spermatozoa collected from the cauda epididymis can efficiently fertilize eggs in vitro [1], male mice ejaculate spermatozoa into the female reproductive tract with accessory gland secretions (e.g. seminal vesicle, prostate, coagulating gland) in vivo. The majority of accessory gland secretions are produced from seminal vesicles in many species (e.g. human, bull) [2, 3]. As mice without seminal vesicles show subfertility [4], seminal vesicle secretions (SVSs) are thought to play a beneficial role in fertilization in vivo.

As one of the physiological functions of the SVSs, copulatory plug formation is well known in several primates (such as chimpanzees) and rodents [5, 6]. For copulatory plug formation, transglutaminase 4 (TGM4), a protein from the coagulating gland and prostate, catalyzes the formation of ϵ-(γ-glutamyl)lysine cross-bridges between some SVS proteins [6–14]. In fact, *Tgm4*-disrupted males show a plug formation defect [15]. Thus, the interaction between SVSs and proteins from the coagulating gland and prostate are required for copulatory plug formation. Mangels et al. showed that copulatory plugs prevent subsequent mating with other males [16], and their previous work suggested that the copulatory plug is involved with sperm release, sperm transition, and coital stimulation, but the critical role remained to be determined [17]. Further, other results showed that the attachment of SVSs to cauda epididymal spermatozoa protects spermatozoa from the immunological defense mechanism of the uterus and also prevents premature capacitation, leading to increased sperm viability and fertilizing ability in vivo [18–20]. Also, SVSs are reported to influence gene expression in the female reproductive tract and subsequent fetal development [21–23]. These results suggest that SVSs have several physiological functions, but the details remain to be elucidated.

In 1987, Chen et al. found seven major proteins in mouse SVSs (SVS1–SVS7) by India ink staining after SDS-PAGE [24]. SVS1 to 3 are substrates for the copulatory plug formation-related enzyme “TGM4” [6–14], and genes encoding SVS2 to SVS6 evolved by gene duplication and formed a cluster on chromosome 2 [25, 26], suggesting redundant functions of SVS1 to SVS6 during plug formation. However, *Svs2* single knockout (−/−) males hardly make copulatory plugs, leading to male subfertility [20]. In addition, SVS4 to SVS6 are not substrates of TGM4 [9] and are expected to play distinct roles, such as in capacitation inhibition [27] and serine protease inhibition [25]. Therefore, SVS1 to SVS6 may share some roles in plug formation, but the importance of each gene remains to be determined.

The remaining SVS, SVS7, is encoded by the *Pate4* (also known as *Pate-B* and *Caltrin*) gene on chromosome 9. *Pate4* mRNA was reported to be expressed not only in the seminal vesicles but also in the testes, epididymides, and prostates by RT-PCR [28, 29]. However, the later study showed that *Pate4* mRNA is expressed not in testis and epididymis but in the prostate and seminal vesicle by northern blotting, and that the signal intensity of *Pate4* mRNA in seminal vesicles is four times higher than in the prostates by qPCR [30]. Thus, the expression of mouse *Pate4* in tissues remains unclear. PATE4 was localized to the head and flagella of mouse epididymal spermatozoa incubated in SVS7 solution [31]. It was found that Ca^2+^-dependent events relating to sperm fertilizing ability, such as in sperm capacitation, sperm motility, the acrosome reaction, and sperm-egg interaction, were regulated by PATE4 in vitro [26, 31–33]. Heckt et al. performed phenotypic analyses of *Pate4*−/− mice [30], but the detailed role of PATE4 in copulatory plug formation and sperm-fertilizing ability remains to be revealed. Here, we examined the physiological function of PATE4 in male fecundity using knockout mice.

## Materials and methods

### Animals

All mice used in this study were purchased from Japan SLC or CLEA Japan. Mice were acclimated to a 12-h light/12-h dark cycle. All animal experiments were approved by the Animal Care and Use Committee of the Research Institute for Microbial Diseases, Osaka University, Japan (#Biken-AP-H25-02 and #Biken-AP-H30-01).

### Sample collection

The testis, epididymis (caput, corpus, and cauda regions), coagulating gland (also known as anterior prostate), prostate (mixture of dorsal, lateral, and ventral regions), seminal vesicle, ovary, and uterus were collected from adult C57BL/6NCr and B6D2F1 mice. SVSs were obtained by squeezing the seminal vesicle immediately after euthanasia. The copulatory plug was collected from ICR males treated with vasoligation within 2 h of mating. These samples were fractured in TRIzol (Ambion) or in Tris-Buffered Saline with Triton-X {50 mM NaCl (Nacalai), 10 mM Tris-HCl (Nacalai), 1% (v/v) Triton-X 114 (Sigma), pH 7.5} containing 1% (v/v) protease inhibitor cocktail (Nacalai), and then used to detect PATE4 in various organs at mRNA and protein levels. Cauda epididymal spermatozoa were dispersed in TYH drops [34] for 5 min. The intrauterine fluid before and 1 h after mating with B6D2F1 males was collected by PBS perfusion (200 μL/each uterine horn). These samples were used for western blotting.

### RT-PCR for tissue expression analysis

The total RNA was reverse-transcribed to cDNA using a SuperScript III First-Strand Synthesis System for RT-PCR (Invitrogen). PCR conditions with primer sets {*Pate4*: 5′-atgaattcagtgacgaaaatcagcacactg-3′ (forward), 5′-ctagaagctattacacaagtttttttcgcagc-3′ (reverse), *Actb*: 5′-catccgtaaagacctctatgccaac-3′ (forward), 5′-atggagccaccgatccaca-3′ (reverse), *Tgm4*: 5′-aatgctgctgcccaccacacat-3′ (forward), 5′-tcaaactgaccaaaggtccagggct-3′ (reverse)} and KOD DNA Polymerase (KOD-Fx neo, TOYOBO) were 94°C for 3 min, denaturing at 94°C for 30 s, annealing at 65°C for 30 s, elongation at 72°C for 30 s for 35 cycles total, followed by 72°C for 2 min.

### Western blotting

Before SDS-PAGE, all samples were mixed with sample buffer containing β-mercaptoethanol [35] and then boiled. A rabbit polyclonal antibody was raised against mouse PATE4 (Accession #: NP_064660.2, amino acid #52 to 69). A goat polyclonal antibody for mouse TGM4 was purchased from Santa Cruz Biotechnology (sc-55787). A monoclonal antibody for SLC2A3 (KS64-10), an abundant protein in the sperm tail, was generated in our laboratory [36]. The horseradish peroxidase (HRP)-conjugated goat anti-rabbit immunoglobulin G (IgG), HRP-conjugated goat anti-rat IgG, and HRP-conjugated bovine anti-goat IgG antibodies were purchased from Jackson ImmunoResearch Laboratories (also see Supplemental Table S1). The PVDF membrane was treated with Tris-Buffered Saline with Tween-20 {TBST, 50 mM NaCl, 20 mM Tris-HCl, and 0.05% (v/v) Tween-20 (Nacalai), pH 7.5} containing 10% skim milk (Becton Dickinson and Company) for 1 h, followed by the primary antibody (1:1000) for 3 h or overnight. After washing, the membrane was treated with the secondary antibody [1:5000 (PATE4 and SLC2A3), 1:50,000 (TGM4)] for 1 h. The HRP activity was visualized with ECL Prime (BioRad). The signal intensity was measured by ImageQuant TL software (GE Healthcare).

### Production of *Pate4* mutant mice with International Mouse Phenotyping Consortium vector

A targeting vector for *Pate4* was purchased from International Mouse Phenotyping Consortium (IMPC) (Project ID: #36005), and subsequently electroporated into EGR-G101 ES cells (http://cell.brc.riken.jp/en/, cell# AES0182) [37] after linearization. PCR conditions to select the correctly targeted ES cells using KOD-Fx (TOYOBO) and primers {5′ arm for *Pate4*: 5′-cagaggcttctctaacatagactac-3′ (forward), 5′-cacaacgggttcttctgttagtcc-3′ (reverse), 3′ arm for *Pate4*: 5′-atccgggggtaccgcgtcgag-3′ (forward), 5′-ctcagtcttccattgattggagcac-3′ (reverse)} were 94°C for 3 min, denaturing at 94°C for 30 s, annealing at 65°C for 30 s, elongation at 72°C for 6 and 4 min (for 5′ arm and 3′ arm, respectively) for 40 cycles total, followed by 72°C for 2 min. ES cells were injected into 8-cell embryos collected from ICR females. These embryos were transplanted into the uterus of pseudo-pregnant ICR recipients. The chimeric males were mated with B6D2F1 females, and then germ-line transmission and genotyping were confirmed by PCR. PCR conditions with primer sets for genotyping {*WT* allele: 5′-caaaacctggtgaatcatgc-3′ (forward), 5′-cgagcacatttgctttgagt-3′ (reverse), *KO* allele: 5′-atccgggggtaccgcgtcgag-3′ (forward), 5′-cgagcacatttgctttgagt -3′ (reverse)} and Taq polymerase (Ex Taq, Takara) were 94°C for 1 min, denaturing at 94°C for 30 s, annealing at 65°C for 30 s, elongation at 72°C for 30 s for 40 cycles total, followed by 72°C for 7 min. The *Pate4* mutant mice with B6D2 congenic background were used for all experiments in this study. Frozen spermatozoa from *Pate4*-disrupted males (B6D2; B6-*Pate4 *< tm1a(KOMP)Osb>, BRC#09990) will be available through RIKEN BRC (http://en.brc.riken.jp/index.shtml).

### Surgical removal of the accessory glands

B6D2F1 males (8-week-old) were used for the removal of accessory glands, as described previously [38]. Four weeks after surgeries, these males were used for the following experiments.

### Count, morphology, motility, and in vitro fertilization of cauda epididymal spermatozoa

Protein extracts from the entire cauda epididymis were subjected to western blotting, and then the signal intensity of SLC2A3 was measured by ImageQuant TL software to estimate the sperm count within the cauda epididymis. Cauda epididymal spermatozoa were dispersed in PBS (for sperm morphology) or TYH drops {for sperm motility and in vitro fertilization (IVF)}. After an incubation period of 120 min, the sperm motility pattern was examined using the CEROS sperm analysis system (software version 12.3; Hamilton Thorne Biosciences) [39]. IVF was performed as described previously [40].

### Male fecundity of *Pate4* mutant mice

*Pate4* mutant males (9- to 17-week-old) were mated with two B6D2F1 females (8- to 11-week-old) for 1.6–4.5 months (see Supplemental Table S2). Sham, coagulating gland removed {CG (−)}, and seminal vesicle removed {SV (−)} males (20- to 27-week-old) were mated with one or two B6D2F1 females (7- to 10-week-old) for 1.5 to 2.0 months (see Supplemental Table S2).

### Collection of copulatory plugs and uterine sperm counts

Pregnant mare serum gonadotropin (PMSG) (5 units, ASKA Pharmaceutical) was injected into the abdominal cavity of B6D2F1 females, followed by human chorionic gonadotropin (hCG) (5 units, ASKA Pharmaceutical) 48 h after PMSG. Twelve hours after hCG, the hormone-treated females were caged with the *Pate4* mutant, sham, CG (−), and SV (−) males (9- to 37-week-old) under observation. Immediately after mating, the existence of the copulatory plugs was checked, and then the leaked semen was collected with pipettes. Within 2 h of mating, the copulatory plug and the uterus were obtained after euthanasia. The spermatozoa in the uterus were collected by PBS perfusion (200 μL/each uterine horn), and then the total sperm count was examined with a hemocytometer.

### Sequential mating

After 11 h of hCG injection, the hormone-treated females were caged with *Pate4*−/− males (9- to 15-week-old) under observation. After successful mating, the females were re-caged with *Pate4*+/+ males (9- to 15-week-old) under observation. After 6–9 h of mating with *Pate4*+/+ males, we collected eggs from the oviducts to observe two pronuclei and then incubated them for 4 days in KSOM drops. KSOM contains 95.0 mM NaCl, 2.49 mM KCl, 0.353 mM KH_2_PO_4_, 0.199 mM MgSO_4_ • 7H_2_O, 2.36 mL 60% (w/w) DL-lactic acid, 0.250 mM pyruvic acid, 0.200 mM glucose, 1.00 g/L BSA, 10.0 μM EDTA • 4Na, 25.0 mM NaHCO_3_, 1.71 mM CaCl_2_ • H_2_O, 10.0 mL/L essential amino acid (Gibco), 5.00 mL/L nonessential amino acid (Gibco), 50 IU/L penicillin (Gibco), 50 μg/L streptomycin (Gibco), 1.00 mM glutamine, and 1.66 mL/L 0.6% phenol red (Daiichi-Sankyo) in deionized and distilled water (unless otherwise noted, we used regents from Sigma). The genome was extracted from each egg at morula and blastocyst stage with 1 μL lysis buffer {20 mM Tris-HCl (pH 8.0), 5 mM EDTA (Nacalai), 400 mM NaCl, 0.3% SDS (Nacalai), and 200 μg/mL Actinase E solution (Kaken pharmaceutical)}. PCR conditions with primer sets for genotyping and KOD-Fx neo were 94°C for 3 min, denaturing at 94°C for 30 s, annealing at 65°C for 30 s, elongation at 72°C for 30 s for 40 cycles total, followed by 72°C for 2 min.

### Modified AI method

We modified the conventional AI method [41, 42], by using a gel loading tip (cat#010-Q, BMBio) for sperm injection (Supplemental Figure S2A) and petroleum jelly for plugging (Nacalai) (Supplemental Figure S2B). AI was performed under Isoflurane anesthesia (Mylan Inc.). After inserting 10–15 mm of the tip (Supplemental Figure S2B), about 50 μl of a sperm suspension was injected into the uterine body. Then, petroleum jelly at the end of the tip was injected to form the artificial plug around the uterine cervix using a plunger (Terumo) (Supplemental Figure S2C).

### Sperm injection by AI

The SVSs of B6D2F1 and *Pate4*−/− males were suspended in 200 μL TYH or PBS, and subsequently centrifuged at 2000–5000 *g* for 5 min, as described previously [20]. Ten microliter of the supernatant was diluted in 90 μL TYH or PBS. Cauda epididymal spermatozoa of B6D2F1 and *Pate4*−/− males were dispersed in 100 μL TYH or PBS drops with and without SVSs for 10–40 min. The uterus of hormone-treated females was artificially injected with 0.08 to 8.8 × 10^6^ cauda epididymal spermatozoa.

### Sperm viability

Thirty minutes after AI, the uterine fluid was collected by TYH or PBS perfusion (200 μL/each uterine horn). The motility of spermatozoa in the middle layer of the drop was recorded using an Olympus BX-53 microscope equipped with a high-speed camera (HAS-L1, Ditect). Spermatozoa that showed flagellar beating were counted.

### In vivo fertilization rates

Seven to fourteen hours after AI, the cumulus of collected eggs was removed with Hyaluronidase (300 μg/mL final conc., Sigma). The number of eggs with two pronuclei was counted.

### Pregnancy rates and fetal development

After 19 days, offsprings were obtained by natural birth or Caesarean section.

### Statistical analyses

All values are shown as the mean ± SD of at least three independent experiments. Statistical analyses were performed using the Student's *t*-test (for Figures 2E and F, and 5B), Mann–Whitney U-test (for Figures 2E, and 5A and D), Steel-Dwass test (for Figures 3B, E, and F, and 4C and D), and Tukey–Kramer method (for Figures 3A, C and D, and 5E), after examining the normal distribution and variance.

### Alignment analysis

The amino acid sequences for SVS1 (accession #AAI25636), SVS2 (accession#: AAI07276), SVS3 (accession#: AAI31999), and PATE4 (accession#: AAI20767) were used for alignment analysis with Clustal omega (https://www.ebi.ac.uk/Tools/msa/clustalo/). The presence and location of the signal peptide were predicted with SignalP 4.1 Server (http://www.cbs.dtu.dk/services/SignalP/).

## Results

### Detection of PATE4

**Figure 1.**
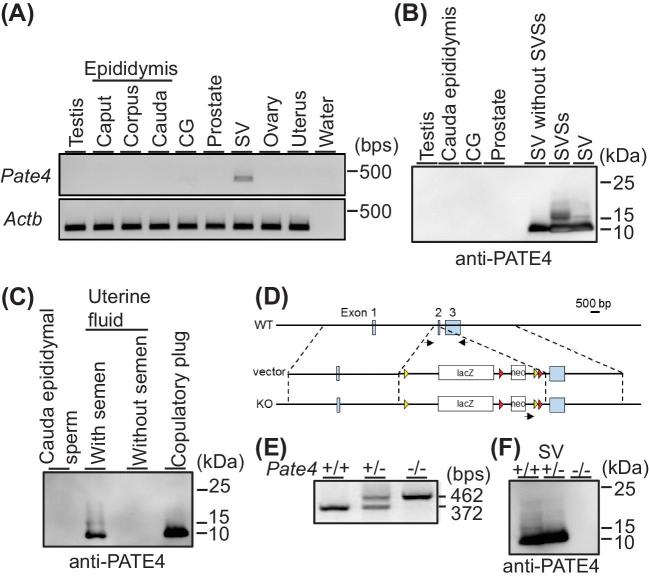
Detection of PATE4 and production of *Pate4* mutant mice. (A) Expression analysis of *Pate4* mRNA by PCR. Actin beta (Actb) was used as the control. CG: coagulating gland, SV: seminal vesicle. (B and C) Detection of PATE4 by western blotting (WB). SVSs: seminal vesicle secretions. (D) Targeting strategy to produce *Pate4* mutant mice. Black arrows show the primer set used for genotyping. Yellow and red triangles show loxP and FRT sites, respectively. neo: neomycin resistance cassette. (E) Genotyping of *Pate4* mutants by PCR. (F) Detection of PATE4 in the seminal vesicle of *Pate4* mutants by WB.

When we performed RT-PCR with cDNAs of male and female reproductive organs, *Pate4* was strongly detected in the seminal vesicle (Figure 1A). Further, we generated an anti-PATE4 antibody and performed western blotting analysis. While we could not detect any signals in the testis, cauda epididymis, coagulating gland, and prostate, a firm doublet signal was detected in seminal vesicles (Figure 1B). To examine whether PATE4 is ejaculated into semen, we collected uterine fluid 1 h after copulation. Though we could not detect PATE4 in the uterus fluid before coitus, PATE4 was detected after coitus in uterine fluid which also contained semen and the copulatory plug (Figure 1C). These results indicate that PATE4 is abundantly secreted from seminal vesicles and then ejaculated into the female reproductive tract.

### Male fecundity of *Pate4*−/− mice

**Figure 2.**
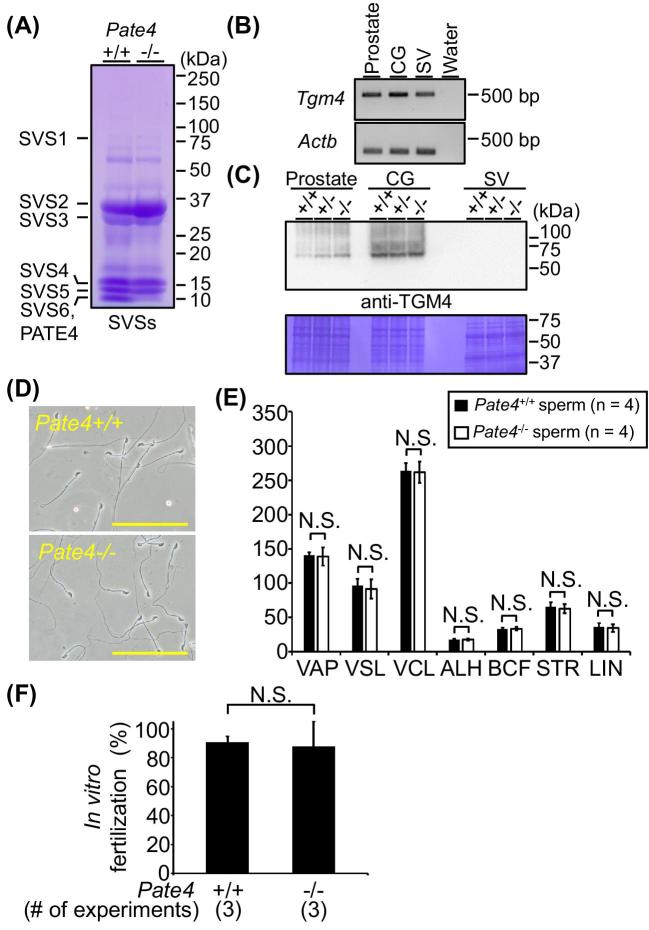
Characteristics of seminal vesicles, prostates, and cauda epididymal spermatozoa in *Pate4*−/− males. (A) Detection of SVS1 to SVS6 and PATE4 in SVSs by Coomassie Brilliant Blue (CBB). (B) Detection of transglutaminase 4 (*Tgm4*) by RT-PCR. (C) Detection of TGM4 by WB. The membrane was stained by CBB (lower panel). (D) Sperm morphology. Scale bars show 100 μm. (E) Sperm motility assay using computer-assisted sperm analysis. After 2 h of incubation, there were no differences in the sperm parameters between *Pate4*+/+ and *Pate4*−/− spermatozoa. VAP: average path velocity (*P* = 0.79), VSL: straight line velocity (*P* = 1.00), VCL: curvilinear velocity (*P* = 0.81), ALH: amplitude of lateral head (*P* = 0.56), BCF: beat cross frequency (*P* = 0.86), STR: straightness of trajectory (*P* = 0.77), LIN: linearity (*P* = 0.77). (F) In vitro fertilization rates. The fertilizing ability of *Pate4*−/− spermatozoa was comparable to *Pate4*+/+ spermatozoa (*P* = 0.79). N.S.: not significant.

To reveal the function of PATE4 in vivo, we generated mice lacking *Pate4* with a targeting vector from IMPC (Figure 1D). Mice were genotyped by genomic PCR (Figure 1E). The breeding pairs of *Pate4* heterozygotes resulted in the predicted Mendelian ratio in offspring {n = 9, wild type (+/+): heterozygous (+/−):−/− = 22 pups (30.1%): 31 pups (42.5%): 20 pups (27.4%)}, and *Pate4*−/− mice grew normally to sexual maturity. As expected from RT-PCR analysis (Figure 1A), *Pate4*−/− females are fertile (n = 5, No. of litters/female/month of caging: 1.16 ± 0.20). While there were no differences in the morphology (Supplemental Figure S1A) and weight (Supplemental Figure S1B) of seminal vesicles, PATE4 protein was not detectable from the seminal vesicles of *Pate4*−/− mice (Figure 1F). SVS1 to SVS6 and TGM4 were detected in wild-type (WT) and *Pate4*−/− males at comparable levels (Figure 2A–C). As we expected from the western blot analysis, *Pate4*−/− cauda epididymal spermatozoa did not show any defects in sperm parameters in vitro such as morphology (Figure 2D), motility parameters (Figure 2E), and in vitro fertilizing ability (Figure 2F).

**Figure 3.**
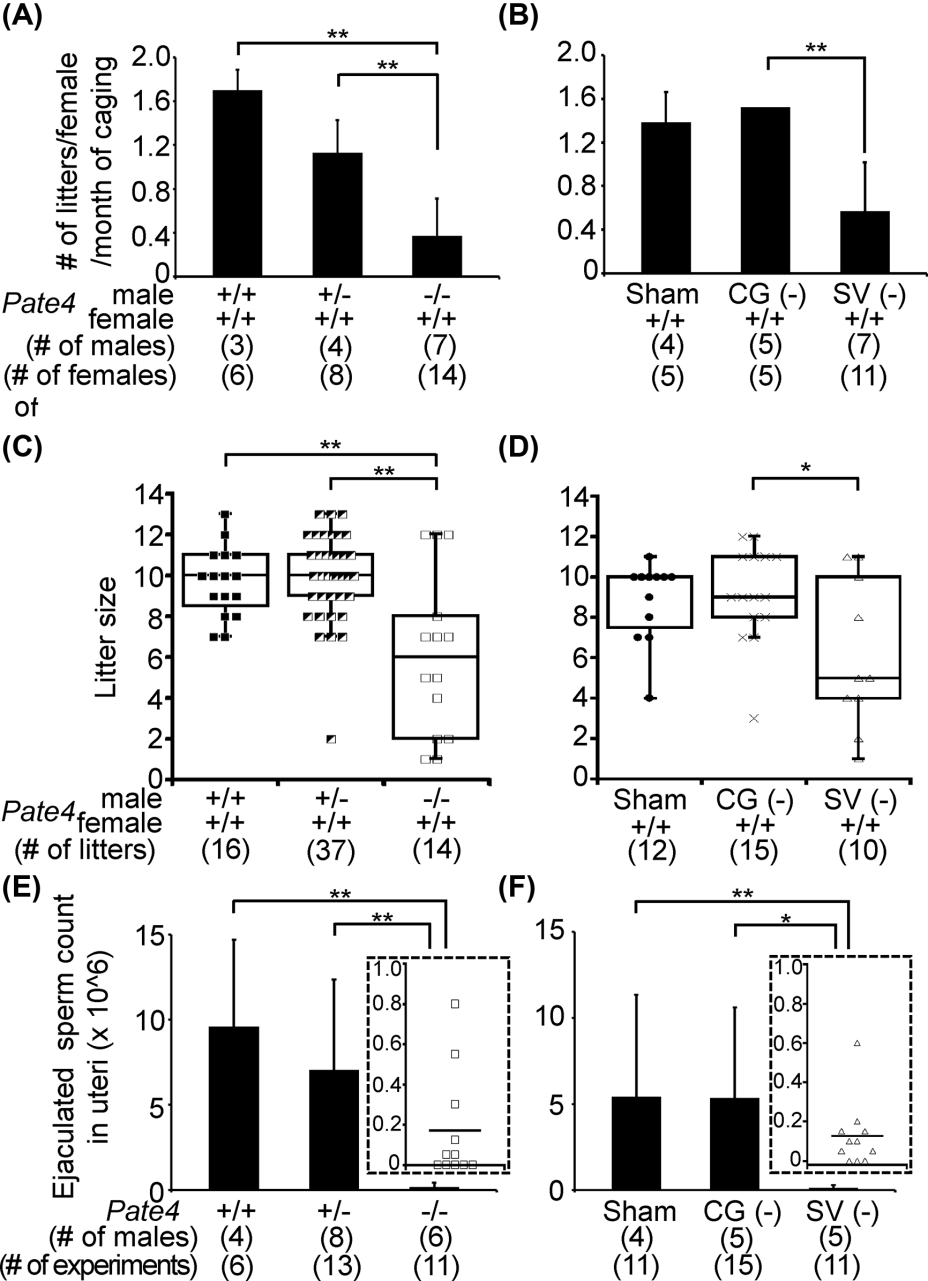
Male fecundity of *Pate4*−/− and SV (−) mice and sperm counts in the uterus. (A and B) No. of litters/female/month of caging. Sham-operated males were used as the control (also see the Supplemental Table S2). CG (−): coagulating gland removal, SV (−): seminal vesicle removal. (C and D) Litter size. Also, see the Supplemental Table S2. (E and F) Ejaculated sperm counts within the uterus. Marks in the inserted figures (□ and ▵) show the sperm count (×10^6^) collected from each female mated with *Pate4*−/− and SV (−) males. *P < 0.05, **P < 0.01.

To characterize the sperm fertilizing ability in vivo, we caged B6D2F1 females with *Pate4* mutant males for 1–5 months. *Pate4*−/− males mated normally, but the pregnancy rate was significantly reduced, compared with *Pate4*+/+ and *Pate4*+/− males (no. of litters/female/month of caging, *Pate4*+/+: 1.70 ± 0.18, *Pate4*+/−: 1.13 ± 0.30, *Pate4*−/− : 0.37 ± 0.34) (Figure 3A; Supplemental Table S2). It should be noted that the average litter size was lower with *Pate4*−/− males {*Pate4*+/+: 9.69 ± 1.70, *Pate4*+/−: 9.95 ± 2.17, *Pate4*−/−: 6.07 ± 3.95 (Figure 3C, and Supplemental Table S2)}.

To further characterize male fecundity, we collected uterine spermatozoa 2 h after mating. While the total sperm count stored in the cauda epididymis of *Pate4*−/− males was comparable (*Pate4*+/+: 3.00 ± 1.85 × 10^7^ spermatozoa, *Pate4*+/−: 2.63 ± 4.00 × 10^7^ spermatozoa, *Pate4*−/−: 2.38 ± 5.33 × 10^7^ spermatozoa), the number of ejaculated spermatozoa collected from the uterus was significantly reduced when compared with the control males {*Pate4*+/+: 9.61 ± 5.07 × 10^6^ spermatozoa, *Pate4*+/−: 7.04 ± 5.32 × 10^6^ spermatozoa (Supplemental Movie S1), *Pate4*−/−: 0.17 ± 0.27 × 10^6^ spermatozoa (Supplemental Movie S2)} (Figure 3E). These results indicate that PATE4 plays a role in male fecundity.

### Phenotypic analysis of *Pate4*−/− males

**Figure 4.**
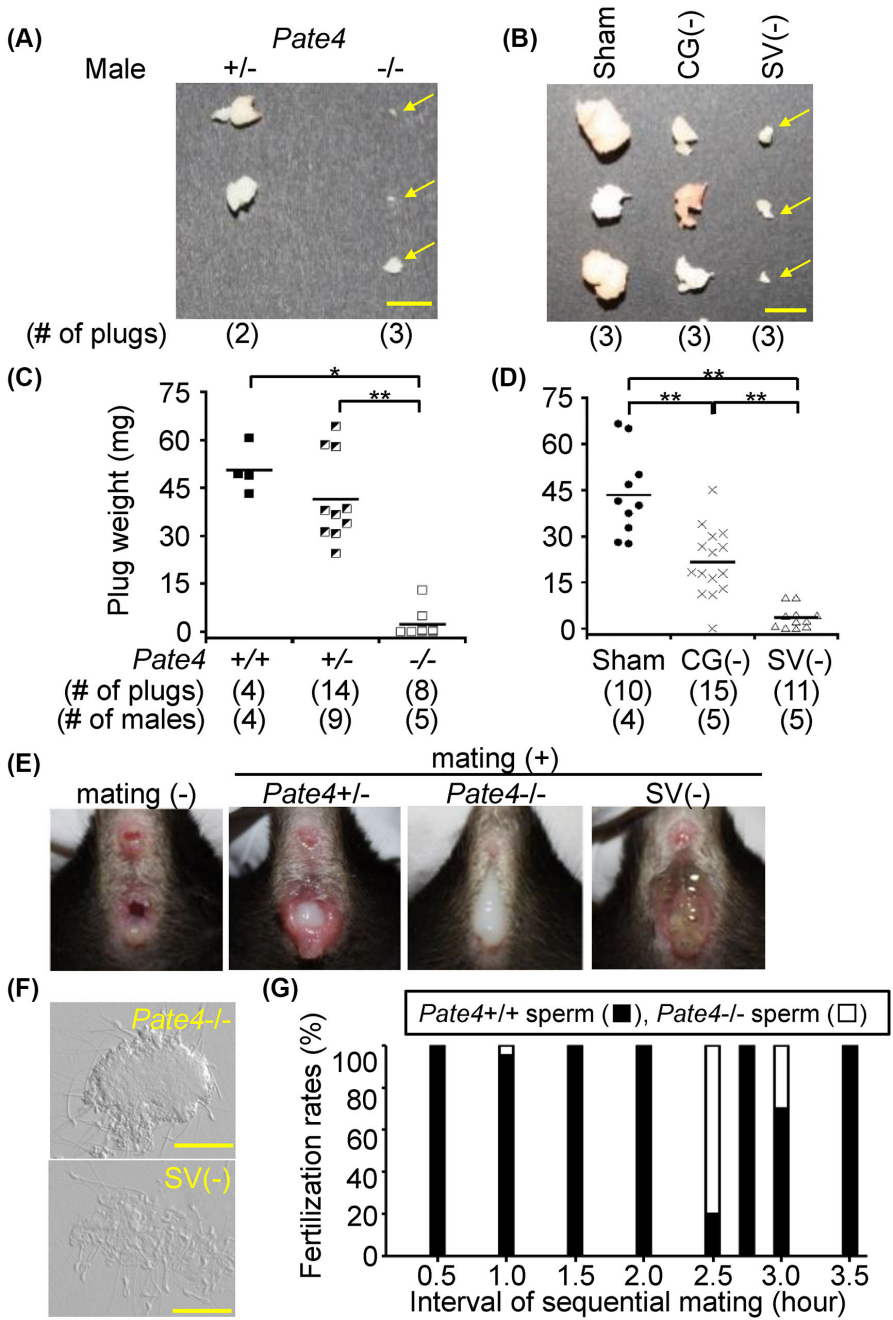
Copulatory plugs and semen leakage of *Pate4*−/− and SV (−) males, and sequential mating. (A and B) Morphology of the copulatory plugs. Yellow arrows show the copulatory plugs of *Pate4*−/− and SV (−) males. The spermatozoa were observed in the uterus of a female mated with these males. Scale bars indicate 5 mm. (C and D) Weight of the copulatory plugs. The copulatory plugs were collected from WT females mated with *Pate4* mutant males (9- to 19-week-old) and surgically treated males (15- to 26-week-old). *P < 0.05, **P < 0.01. (E) Observation of the vaginal opening before and immediately after mating. The ejaculated leakage was observed in females mated with *Pate4*−/− and SV (−) males. (F) The leakage contained spermatozoa. Scale bars represent 50 μm. (G) Fertilization rates obtained by sequential mating. After 0.5–3.5 h of mating with *Pate4*−/− males, the females were sequentially mated with *Pate4*+/+ males.

When we observed copulatory plug formation every 30 min during the mating experiment, we found that *Pate4*−/− males produce significantly smaller plugs compared with *Pate4*+/+ and *Pate4*+/− males (*Pate4*+/+: 50.48 ± 7.31 mg, *Pate4*+/−: 40.55 ± 13.60 mg, *Pate4*−/−: 2.37 ± 4.61 mg) (Figure 4A and C). It should be noted that there was no difference in the amount of copulatory plug-related proteins SVS1 to SVS3 and TGM4 [6–15, 20], between WT and *Pate4*−/− males (Figure 2A and C). These results indicate that PATE4 is critical for copulatory plug formation.

To understand the effect of the decreased copulatory plug size, we observed the vaginal opening immediately after copulation. We found that the semen leaked out of females mated with *Pate4*−/− males (Figure 4E and F). With the finding that fewer spermatozoa were found in females mated with *Pate4*−/− males (Figure 3E), these results indicate that the copulatory plug has a role in keeping ejaculated spermatozoa in the uterus.

### Fecundity and phenotypical analysis of coagulating gland- and seminal vesicle- removed males

Some SVS proteins are required for the copulatory plug formation and sperm fertilizing ability [6–14, 20, 27, 38, 43]. We examined whether the lack of multiple factors including PATE4 from the seminal vesicles led to a more severe decrease in male fecundity using males with their seminal vesicle surgically removed {SV (−)} (Supplemental Figure S1C). We used the sham-operated and coagulating gland-removed {CG (−)} males as the control for plug formation. The fecundity of CG (−) males was comparable to the sham-operated males, but the pregnancy rate of females caged with SV (−) males was reduced {no. of litters/female/month of caging, Sham: 1.39 ± 0.27, CG (−): 1.53 ± 0.00, SV (−): 0.57 ± 0.45} (Figure 3B; Supplemental Table S2). Further, the average litter size largely varied between SV (−) males {Sham: 8.83 ± 1.99, CG (−): 9.13 ± 2.39, SV (−): 6.10 ± 3.67 (Figure 3D; Supplemental Table S2)}. The sperm count in the cauda epididymis of SV (−) males was normal {Sham: 1.83 ± 0.12 × 10^7^ spermatozoa, CG (−): 1.40 ± 0.27 × 10^7^ spermatozoa, SV (−): 1.54 ± 0.07 × 10^7^ spermatozoa}, but the ejaculated sperm count observed in the uterus after mating with SV(−) males was much lower than the sham-operated and CG (−) males {Sham: 5.45 ± 5.89 × 10^6^ spermatozoa (Supplemental Movie S3), CG (−): 5.38 ± 5.23 × 10^6^ spermatozoa (Supplemental Movie S4), SV (−): 0.13 ± 0.17 × 10^6^ spermatozoa (Supplemental Movie S5)} (Figure 3F). As we expected from the function of seminal vesicles, SV (−) males hardly make a copulatory plug {Sham: 43.52 ± 13.80 mg, SV (−): 3.53 ± 3.59 mg} (Figure 4B and D), leading to semen leakage (Figure 4E and F). The copulatory plug of CG (−) males was half of the sham-operated males {Sham: 43.52 ± 13.80 mg, CG (−): 21.55 ± 11.19 mg} (Figure 4B and D), but we could not observe semen leakage. Thus, as the phenotype of *Pate4*−/− males is similar to that of SV (−) males, PATE4 functions as the major factor in seminal vesicle secretions.

### Sequential mating with *Pate4*−/− males

When females were re-caged with *Pate4*+/+ males immediately after mating with *Pate4*−/− males, these females succeeded in subsequent mating. Thus, we examined which spermatozoa were fertilizing eggs. As shown in Figure 4G, within the 2-h mating interval, almost all fertilized eggs were from *Pate4*+/+ spermatozoa (n = 4, fertilization rate, *Pate4*+/+: 98.8 ± 2.4%, *Pate4*−/−: 1.2 ± 2.4%). Further, even in intervals greater 2 h, the fertilization rates of *Pate4*+/+ spermatozoa were higher than the *Pate*4−/− spermatozoa (n = 4, fertilization rates, *Pate4*+/+: 72.6 ± 37.7%, *Pate4*−/−: 27.4 ± 37.7%). These results indicate that the copulatory plug inhibits subsequent mating, and has a critical function in the male reproductive strategy.

### In vivo fertilizing ability of *Pate4*−/− spermatozoa

**Figure 5.**
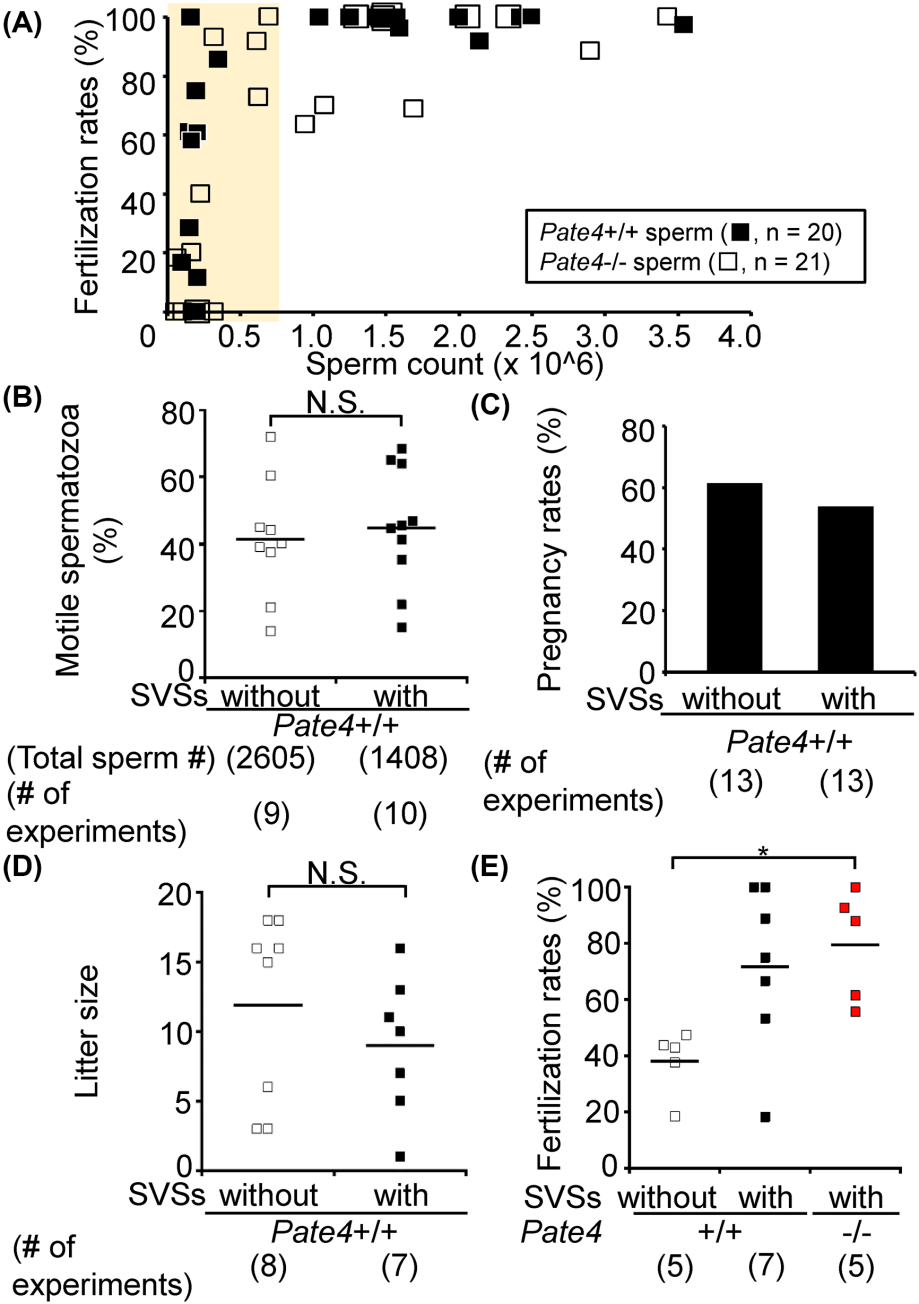
In vivo fertilizing ability of *Pate4*−/− spermatozoa and the effect of *Pate4*−/− SVSs on fertilization rates using AI. (A) Fertilization rates by artificially injecting cauda epididymal spermatozoa. The yellow-colored region shows the sperm count in the uterus of *Pate4*−/− males (see Figure 3E). There are no difference in the fertilizing ability between *Pate4*+/+ and *Pate4*−/− spermatozoa (<10^6^ spermatozoa: *P* = 0.67, ≥10^6^ spermatozoa: *P* = 0.55). (B to D) Effect of *Pate4*+/+ SVSs on AI. The sperm motile rates (B, *P* = 0.69), pregnancy rates (C), and litter size (D, *P* = 0.30) were examined. (E) Effect of *Pate4*+/+ and *Pate4*−/− SVSs on the fertilization rates. Though SVSs have a positive effect on fertilization rates, there was no difference between AI with *Pate4*+/+ and *Pate4*−/− SVSs. *P < 0.05, N.S.: not significant.

To examine the fertilizing ability of *Pate4*−/− spermatozoa in vivo, we collected the cauda epididymal spermatozoa, injected them into the uterus, and sealed the uterine cervix with petroleum jelly {artificial insemination (AI), Supplemental Figure S2A–C}. We consistently obtained high fertilization rates when we used 1 × 10^6^ or more spermatozoa in the insemination fluid {*Pate4*+/+: 98.56 ± 2.70% (197/202 eggs), *Pate4*−/−: 91.91 ± 13.33% (252/276 eggs)} (Figure 5A). However, when we injected less than 1 × 10^6^ spermatozoa, the fertilization rates largely varied {*Pate4*+/+: 49.77 ± 33.73% (123/247 eggs), *Pate4*−/−: 41.60 ± 40.40% (97/262 eggs), Figure 5A}. There were no significant differences in the fertilizing ability between *Pate4*+/+ and *Pate4*−/− cauda epididymal spermatozoa. Thus, *Pate4*−/− spermatozoa have comparable fertilizing ability with WT spermatozoa in vivo.

### Effect of preincubating sperm in PATE4 on AI

To examine the effect of SVSs on sperm fertilizing abilities in vivo, we incubated cauda epididymal spermatozoa in TYH medium with or without SV solutions prior to AI. When we collected spermatozoa 30 min after AI, there were no differences in sperm motility {with *Pate4*+/+ SVSs (1408 spermatozoa, n = 10): 44.86 ± 17.78%, without *Pate4*+/+ SVSs (2605 spermatozoa, n = 9): 41.51 ± 17.68%, Figure 5B}. In addition, there were no differences in pregnancy rates {with *Pate4*+/+ SVSs (n = 13): 53.85%, without *Pate4*+/+ SVSs (n = 13): 61.54%; Figure 5C} and litter size (with *Pate4*+/+ SVSs: 9.00 ± 5.07, without *Pate4*+/+ SVSs: 11.88 ± 6.66; Figure 5D; Supplemental Figure S2D). It is noteworthy that both *Pate4*+/+ and *Pate4*−/− SVSs improved the fertilization rates using 0.8 to 1.8 × 10^5^ spermatozoa {with *Pate4*+/+ SVSs: 71.72 ± 29.26% (131/203 eggs), with *Pate4*−/− SVSs: 79.56 ± 19.76% (95/119 eggs), without *Pate4*+/+ SVSs: 37.97 ± 11.51% (31/99 eggs); Figure 5E}, implicating SVSs might have a positive role when sperm number is low in vivo, but that PATE4 is not the key molecule.

## Discussion

The expression of mouse *Pate4* in tissues remained to be clarified, but here we demonstrated PATE4 abundantly presents in the seminal vesicles at mRNA and protein levels (Figure 1A and B). It is known that *Pate4* is a member of the *Pate* family composed of 13 genes clustered on mouse chromosome 9 [28]. As some parts of the nucleotide sequence of *Pate4* are similar to the other *Pate* family genes, nonspecific binding of primers might cause the discrepancy of RT-PCR results between this study and previous papers.

As we had generated *Pate4*−/− mice before the report of Heckt et al. [30], we used our KO mice for this study. We used the same *Pate4* targeting vector construct from KOMP as the previous report [30], but the genetic background used to maintain the mouse line was different between this study (B6D2 background) and the previous paper (B6J background). The previous paper indicated that fecundity of *Pate4*−/− males were comparable to the control males [30], but we revealed that *Pate4*−/− males were subfertile (Figure 3A and C). When we used seven males of *Pate4*−/− mice for the mating test, the male fecundity varied by individuals (see Supplemental Table S2, no. of litters/female/caging of month, 0–0.96). Thus, the individual differences of *Pate4*−/− males may cause the difference in male fecundity between our study and the previous report. And, our result suggests that the subfertile phenotype may be easily masked by genetic background.

PATE4 was absent from the seminal vesicles of *Pate4*−/− mice (Figure 1F), but the copulatory plug related proteins “SVS1 to SVS3” were detected in *Pate4*+/+ and *Pate4*−/− seminal vesicles at comparable level (Figure 2A). The sequence homology among PATE4, SVS1, SVS2, and SVS3 is low at the amino acid level (Supplemental Figure S3; PATE4 vs SVS1: 23.9%, PATE4 vs SVS2: 23.5%, PATE4 vs SVS3: 17.7%). These results suggest that the remaining factors for plug formation in *Pate4*−/− males cannot compensate for the lack of PATE4. To form the copulatory plug, it is known that TGM (e.g. TGM4) catalyzes the formation of ϵ-(γ-glutamyl)lysine which cross-bridges among SVS1 to SVS3 [6–14]. Lin et al. showed that the peptide sequence “QXK(S/T)” in SVS3 acts as the transglutaminase cross-linking sites by the reaction of guinea pig liver transglutaminase and recombinant polypeptides from SVS3 (Supplemental Figure S3C) [10]. We also found this peptide sequence in SVS2, but the sequence is not conserved in PATE4 (Supplemental Figure S3B). SVS1 also does not contain the sequence “QXK(S/T)”, but Tseng et al. showed that two glutamine residues in SVS1 were the major site for TGM4 crosslinking by mass spectrometry (Supplemental Figure S3A) [8]. As PATE4 also has four glutamine and ten lysine residues (Supplemental Figure S3), our results suggest that PATE4 may have an unidentified target sequence for TGM4 or a function to promote plug formation independent from TGM4.

It is well known that proteins secreted from coagulating glands are required for plug formation [15, 17]. The plug weight from CG (−) males was reduced to almost half of the sham-operated males (Figure 4B and D), but CG (−) males were fertile (Figure 3B), as previously reported [4]. *Tgm4* mRNA was detected in the seminal vesicle, coagulating gland, and prostate (Figure 2B), corresponding with the Unigene database and a previous study [44]. Further, we revealed that the TGM4 protein was detected in the prostate and coagulating gland (Figure 2C). These results indicate that TGM4 localized in both the coagulating gland and the prostate contribute to copulatory plug formation.

Detailed functions of SVSs on sperm-fertilizing ability and fetal development remain to be known. Kawano et al. showed that SVSs improved sperm viability and increased the fertilization rate in vivo [20]. However, with sperm counts comparable to that observed in normal matings (1 × 10^6^ or more), we could not find a significant difference in the rates of sperm motility between spermatozoa with and without SVSs (Figure 5B). Also, 2 h after mating with SV (−) males, the ejaculated spermatozoa in the uterus survived (Supplemental Movie S5). Further, SVSs did not increase the pregnancy rates nor litter size (Figure 5C and D). Given the data presented, SVSs do not appear to play an essential role in sperm-fertilizing ability, implantation, or fetal development in mice.

When we lowered the sperm count in AI, WT SVSs improved fertilization rates, implicating a positive function of WT SVSs on sperm function in vivo. Even in such a case, PATE4 does not contribute to increased sperm-fertilizing ability (Figure 5E). Our results suggest that other components secreted from seminal vesicles support sperm-fertilizing ability in vivo but only when sperm count is low. Many researchers have analyzed the sperm-fertilizing ability using AI, but the fertilization rates varied among papers [20, 41, 42, 45], indicating that an AI method has yet to be standardized. We used females after 12 h of hCG injection for AI, and then obtained the high fertilization rates, corresponding with the previous papers [42]. Thus, for AI with hormone-treated females, the timing after 12 h of hCG injection is considered as the best. Previous studies showed that sperm parameters, such as the morphology, sperm motility, and fertility rate, varied between genetic backgrounds of mouse strains [46, 47]. Thus, the difference between the previous report and this study may be due to these problems.

Here, we revealed that PATE4 is an essential factor for copulatory plug formation. Through phenotypic analysis of *Pate4*−/− males, we found that the copulatory plug has a physiological function to keep the spermatozoa in the uterus, leading to an increase in fertilization rates. Further, females mated with *Pate4*−/− males became pregnant from subsequent matings with *Pate4*+/+ males due to the plug formation defect. Thus, the copulatory plug has dual functions not only to prevent subsequent matings but also to maintain proper sperm count for fertilization in the female reproductive tract, as a winner-take-all strategy to advance male reproduction.

## Supplementary data

**Supplemental Figure S1.** Morphology and weight of *Pate4*−/− seminal vesicles, and the production of the male accessory gland-removed mice. (A) Morphology of seminal vesicles. Scale bars show 5 mm. (B) Seminal vesicle (SV) weight/body weight (BW). There was no difference between *Pate4*+/+ and *Pate4*−/− SVs (*P* = 0.78). N.S.: not significant. (C) Accessory glands were surgically removed, as described previously [38]. CG: coagulating gland.

**Supplemental Figure S2.** Modification of AI and pups produced by AI. (A) Pipette tip for AI. Scale bar shows 5 mm. (B and C) Procedure of AI. Ten to fifteen millimeter of a tip containing the sperm suspension (50 μL) and petroleum jelly for plugging were inserted into the uterus of female mice (B). By inserting 4–5 mm of a plunger into the tip, the sperm suspension was injected into the uterus, and the petroleum jelly formed an artificial plug around the uterine cervix (C). (D) Pups produced from AI. There were no difference in the pregnancy rates and litter size between AI with and without SVSs (also see Figure 5C and D).

**Supplemental Figure S3.** Alignment analysis between PATE4, SVS1, SVS2, and SVS3. (A) Comparison between PATE4 and SVS1. (B) Comparison between PATE4 and SVS1. (C) Comparison between PATE4 and SVS1.

Blue-colored letters: signal peptide; red boxes: potential target sites for TGM4; gray-highlighted regions: fully conserved residue.

**Supplemental Movies 1 and 2**. Observation of *Pate4* mutant mouse spermatozoa in the uterus. *Pate4*+/− (Supplemental Movie S1) and *Pate4*−/− (Supplemental Movie S2) spermatozoa were collected from the uterus by PBS perfusion.

**Supplemental Movies 3–5**. Observation of spermatozoa within the uterus after mating with surgically treated males. Spermatozoa of Sham (Supplemental Movie S3), CG (−) (Supplemental Movie S4), and SV (−) (Supplemental Movie S5) males were collected from the uterus by PBS perfusion.

## Supplementary Material

Supplemental Files

## Notes

Edited by Dr. Monika A. Ward
