# Supplementary material for: Seminal vesicle secretory protein 7, PATE4, is not required for sperm function but for copulatory plug formation to ensure fecundity
Source: Biol Reprod. 2018 Nov 18;100(4):1035–45. doi: 10.1093/biolre/ioy247 (PMC6483057; doi:10.1093/biolre/ioy247)
Supplement: Supplemental Files [file ioy247_supplemental_files.zip › ioy247-Supplemental_Table_S2_Noda_et_al..docx]

| **Supplemental Table S2. Male fecundity of *Pate4*^+/+^, *Pate4*^+/-^, *Pate4*^-/-^, Sham, CG (-), and SV (-) males** | | | | | |
| --- | --- | --- | --- | --- | --- |
| Cage No. | Female  (No. of mice) | Male  (No. of mice) | Months of caging | No. of litters (No. of pups/litter) | No. of litters/female  /month of caging |
| 1 | B6D2F1 (2) | *Pate4*+/+ #1 | 1.6 | 5 (9, 8, 13, 10, 10) | 1.60 |
| 2 | B6D2F1 (2) | *Pate4*+/+ #2 | 1.6 | 6 (7, 9, 11, 10, 12, 9) | 1.92 |
| 3 | B6D2F1 (2) | *Pate4*+/+ #3 | 1.6 | 5 (7, 11, 10, 8, 11) | 1.60 |
| 4 | B6D2F1 (2) | *Pate4*+/- #1 | 4.5 | 13 (10, 2, 11, 13, 7, 8, 12, 11, 12, 9, 12, 9, 12) | 1.43 |
| 5 | B6D2F1 (2) | *Pate4*+/- #2 | 4.5 | 11 (7, 7, 9, 10, 9, 10, 10, 10, 8, 8, 10) | 1.21 |
| 6 | B6D2F1 (2) | *Pate4*+/- #3 | 3.5 | 8 (10, 13, 12, 10, 9, 12, 11, 11) | 1.15 |
| 7 | B6D2F1 (2) | *Pate4*+/- #4 | 3.5 | 5 (11, 11, 11, 13, 8) | 0.72 |
| 8 | B6D2F1 (2) | *Pate4*-/- #1 | 4.3 | 3 (1, 8, 12) | 0.35 |
| 9 | B6D2F1 (2) | *Pate4*-/- #2 | 4.3 | No pup | 0 |
| 10 | B6D2F1 (2) | *Pate4*-/- #3 | 4.5 | 4 (2, 5, 4, 7) | 0.44 |
| 11 | B6D2F1 (2) | *Pate4*-/- #4 | 4.5 | 1 (7) | 0.11 |
| 12 | B6D2F1 (2) | *Pate4*-/- #5 | 4.4 | 1 (2) | 0.11 |
| 13 | B6D2F1 (2) | *Pate4*-/- #6 | 1.6 | 2 (7, 1) | 0.64 |
| 14 | B6D2F1 (2) | *Pate4*-/- #7 | 1.6 | 3 (12, 12, 5) | 0.96 |
| 15 | B6D2F1 (2) | Sham #1 | 1.5 | 3 (10, 7, 10) | 0.98 |
| 16 | B6D2F1 (1) | Sham #2 | 2.0 | 3 (11, 9, 4) | 1.53 |
| 17 | B6D2F1 (1) | Sham #3 | 2.0 | 3 (10, 10, 8) | 1.53 |
| 18 | B6D2F1 (1) | Sham #4 | 2.0 | 3 (7, 10, 10) | 1.53 |
| 19 | B6D2F1 (1) | CG (-) #1 | 2.0 | 3 (7,11, 12) | 1.53 |
| 20 | B6D2F1 (1) | CG (-) #2 | 2.0 | 3 (8, 9, 3) | 1.53 |
| 21 | B6D2F1 (1) | CG (-) #3 | 2.0 | 3 (9, 9, 11) | 1.53 |
| 22 | B6D2F1 (1) | CG (-) #4 | 2.0 | 3 (8, 9, 11) | 1.53 |
| 23 | B6D2F1 (1) | CG (-) #5 | 2.0 | 3 (7, 11, 12) | 1.53 |
| 24 | B6D2F1 (2) | SV (-) #1 | 1.5 | No pup | 0 |
| 25 | B6D2F1 (2) | SV (-) #2 | 1.5 | 3 (4, 8, 2) | 0.98 |
| 26 | B6D2F1 (2) | SV (-) #3 | 1.5 | 3 (5, 5, 1) | 0.98 |
| 27 | B6D2F1 (2) | SV (-) #4 | 1.5 | No pup | 0 |
| 28 | B6D2F1 (1) | SV (-) #5 | 2.0 | 1 (4) | 0.51 |
| 29 | B6D2F1 (1) | SV (-) #6 | 2.0 | 1 (11) | 0.51 |
| 30 | B6D2F1 (1) | SV (-) #7 | 2.0 | 2 (11, 10) | 1.02 |
