# Supplementary material for: Seminal vesicle secretory protein 7, PATE4, is not required for sperm function but for copulatory plug formation to ensure fecundity
Source: Biol Reprod. 2018 Nov 18;100(4):1035–45. doi: 10.1093/biolre/ioy247 (PMC6483057; doi:10.1093/biolre/ioy247)
Supplement: Supplemental Files [file ioy247_supplemental_files.zip › Supplementary_Figures(revised)_Noda et al..pdf]

Supplemental Figure S1 (Noda *et al*.,)

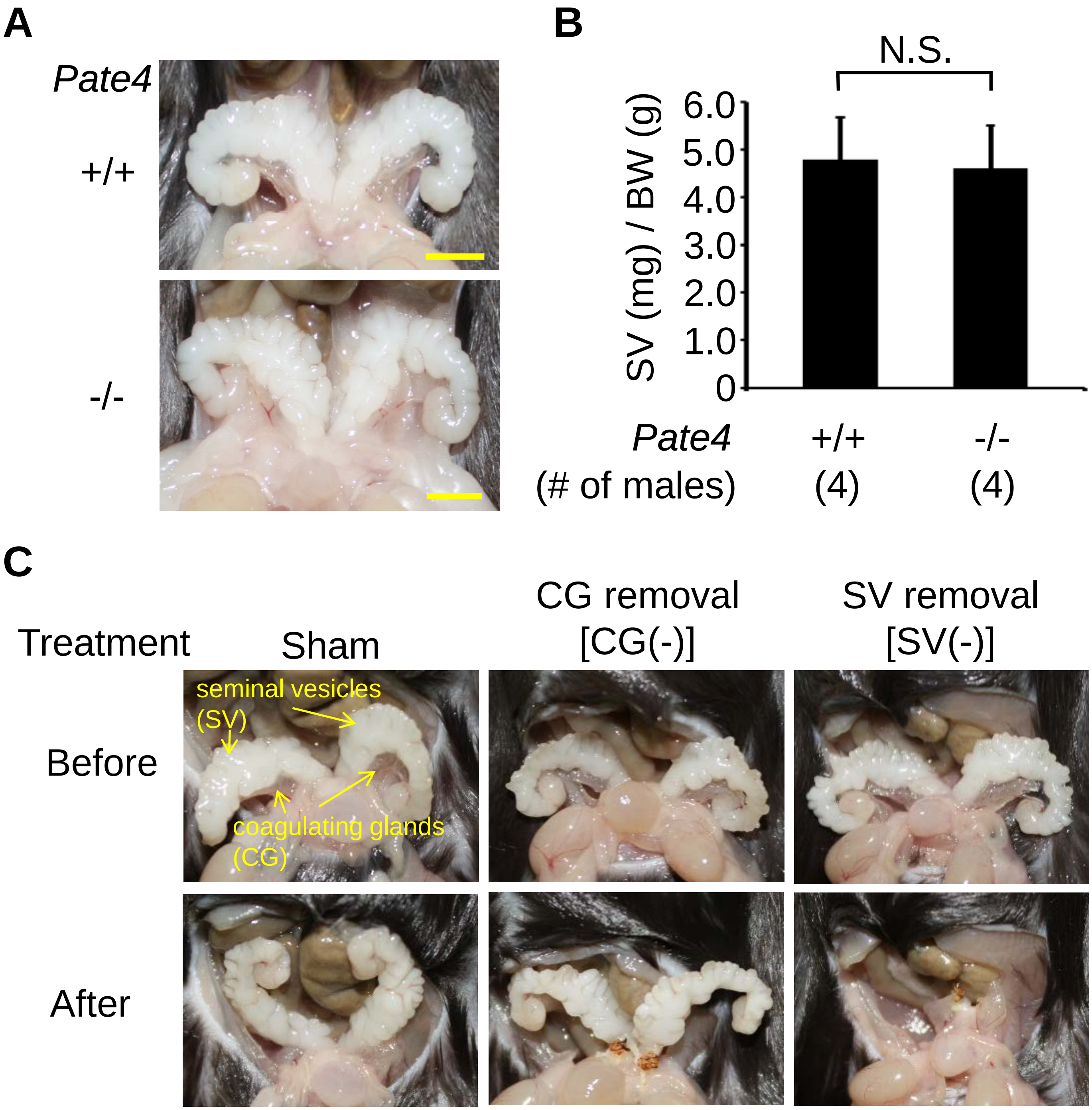

Supplemental Figure S2 (Noda *et al*.,)

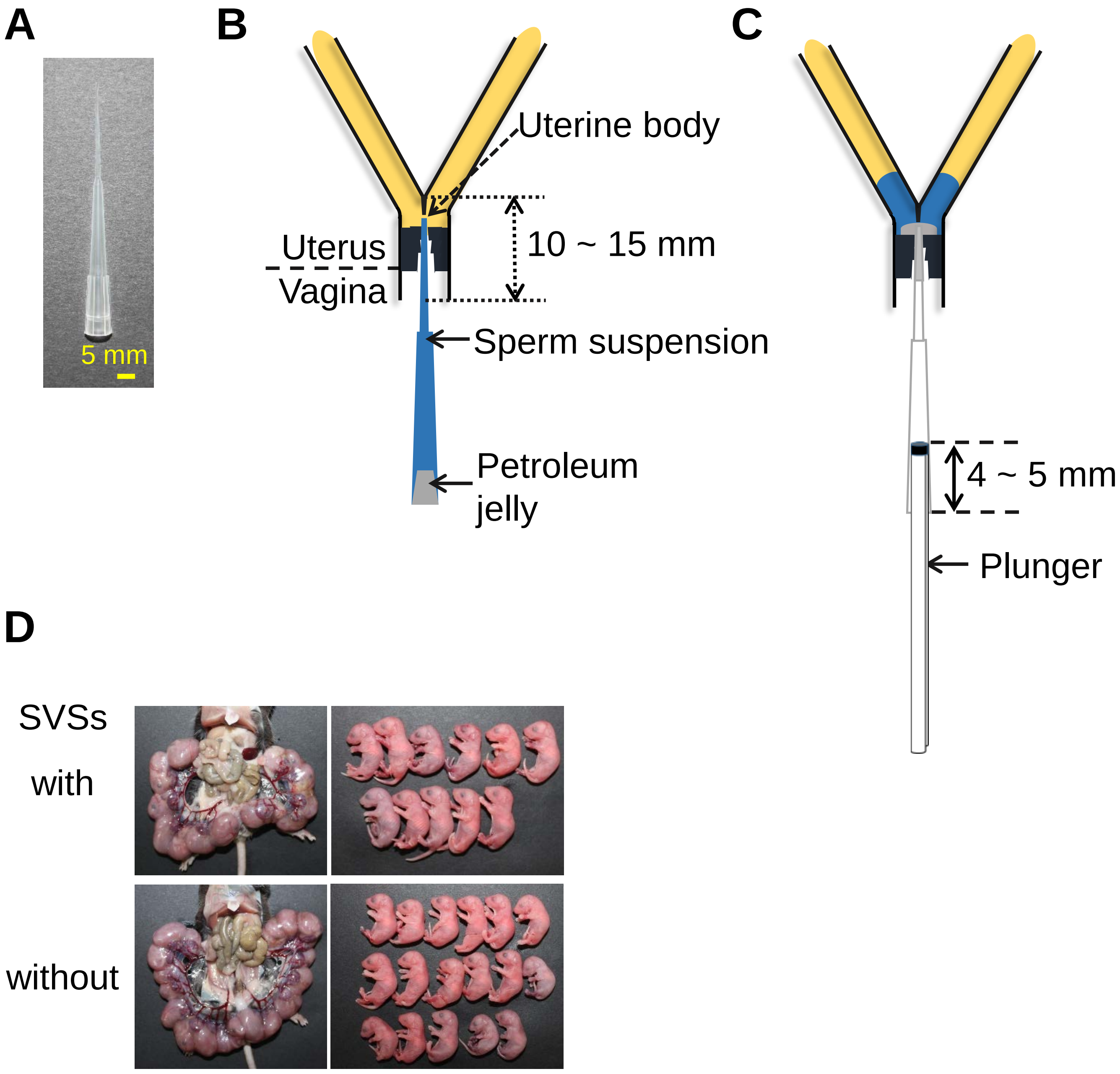

Supplemental Figure S3 (Noda *et al*.,)

A

```
PATE4    ------------------------------------------------------------    0
SVS1     MSLVGRTLVLGWMTTILVVHMSVAKHSRWALRTKPQMLWDLSGEELEAVHNFVMSSKELE    60

PATE4    ----------MNSVTKISTLLIVILSFLCF------------------------------    20
SVS1     LQPSTLLTLAKNSVFLIEMLMPKKYEVLAFLDKGAMLPLREARVLIYFGAQEYPNVTEYA    120

PATE4    -----------------------------------------------------VEGLICN    27
SVS1     VGPVEQPMYMRKLNRKGGQQLSWASRPMSKVESALLLHTLKTATKPLQEFFFDTTGFTLQ    180

PATE4    SCEK--------------------------------------------------------    31
SVS1     DCNGGCLTFTNVGPRAMTFGKRHSWFLLQRIMNDYFLQPTGLEILLDHGSTDVQDWIVEQ    240

PATE4    ------------------------------------------------------------    31
SVS1     VWYNGKFYNSPEELAQKYADGEVDIVVLEDPLPKTTEQFPQFSTYKPYAEFLMPISKGGP    300

PATE4    ------------------------------------------------------------    31
SVS1     RVIQLYPEFPMPISKGGPQKAQPDPKFPIPISKGGPQVAQPYREFPMPISKGGPQVVQPD    360

PATE4    ------------------------------------------------------------    31
SVS1     PKFPIPISKGGPRVPQPHAKFPMPISKGGPRVVQPKAKFPMPISKGGPQIAQPHVPHYRL    420

PATE4    ----------------------------------------------------------SR    33
SVS1     KHNTVLYGDWNFFFKLHSSYGLQLFNVHFRGERIAYEVGVQEVMALYRGHTAAGRKTKYM    480

PATE4    DSRCTMSQSRCVAKPGESCSTVSHFVGTKHVYSKQMCSPQCKEKQLNTGKKLIYIMCCEK    93
SVS1     DVGWGLGGITHQLTPGIDCPHEATFLDAIHYYDSDGPV-------LSRRALCIFEMPLRQ    533

PATE4    ----NLCNSF--------------------------------------------------    99
SVS1     YFNSNFRSSFSSYAKLSGPMLVLRTASTIHNHDYIWDFIFHSNGMMEGKMYATGYVHATF    593

PATE4    ------------------------------------------------------------    99
SVS1     YSSEGLLYHSRLHTHLLGNVHSHLAHYRIDLDVAGTKNRFQTLKMRLENIMDPWSQQVKP    653

PATE4    ------------------------------------------------------------    99
SVS1     ILDKTQYSWERQAAFHFRQTLPKYLLFSNTGKSVSGLSHSYRLHVPSMAEQVLPPGWQTS    713

PATE4    ------------------------------------------------------------    99
SVS1     PAFTWPRYQLAVTKYQESERFHGSLYNQNHHWAYPMVFENFIHNNENIEDEDLVAWVTVG    773

PATE4    -----------------------------------------------        99
SVS1     LSHNHHSEIVPSVATPGNSAGFLLQPFDFYNSFRRYTASPTHAQCVC        820
```

B

```
PATE4    MNSVTKISTLLIVILSFLCFVEGLIC----------------------------NSCEKS    32
SVS2     MKSSVFVLSLLLIL-ERQSAVVGQYGATKGHFQSSSSEGFMLGQKGRLSFGIKGGSDEAA    59

PATE4    RDSRCTMSQSRCVAKPGESCSTVSHFVGTKHVYSKQMCSPQ------CKEK---------    77
SVS2     EESLFMQSQRRVYGQGGGDMTQTRVSQEHTSVKGAALCRNGQVSQLKSQESQIKSYGQVK    119

PATE4    ---QLNTGKKLI-----------------------------YIMCC--------------    91
SVS2     SSGQLKSGGSAFGQVKSSVSQIKSYGQLKSGGQLKSGGPAFGQVKSQESQIKSYGQLKSS    179

PATE4    -------------------------------------------EKNLCNSF---------    99
SVS2     GQLKSGGSAFGQVKSSVSQIKSYGQLKSGGSQVKSYGQTKSYGEEGQLNSFSQLKSQGAQ    239

PATE4    ------------------------------------------------------------    99
SVS2     LKSYGQQKSQQQSSFSQVKSQSSQLKSYGQQKSLKGFSQQTQHKGFAMDEGMSQVRKQFS    299

PATE4    ------------------------------------------------------------    99
SVS2     DDDLSVQQKSTQQMKTEEDLSQFGQQRQYGQERSQSYKGYLEQYRKKVQEQQRKNFNPGN    359

PATE4    ----------------      99
SVS2     YFTKGGADLYQAQLKG      375
```

C

```
PATE4    MNSVTKISTLLIV---------------------------IL------------------    15
SVS3     MKSIFFSLSLLLLLEKKAAGIELYAGGTKGHFLVKTSPLMFIGKNQFLYGHKEEQEEAPE    60

PATE4    ---------------------------SFLCFVEGLICNSCEKSRDSRCTMSQSR-----    43
SVS3     ESIFVQTKHHAYGQDADADMGGALSSQELTSLKEDIVCEEEDELAQQKSQLPSQSQIKSQ    120

PATE4    ---------CVAKPGESC------------------------------------------    52
SVS3     TQVKSYAAQLKSQPGQLKTIGQVKSQTMLKSHGAPLKSFKARLNLREDIPQQVKGRGYGL    180

PATE4    -----------STVS-----------------HFVGTKHVYSK-------QMCSPQCKEK    77
SVS3     AEDLAQVRQQPAKVHRLKGKHRQSRKTAAFYPQFRRHSRPYPRYFVQFQEQLQGSVHHTK    240

PATE4    QLNTGKKLIYIMCCEKNLC----NSF--   99
SVS3     SFYPGPGMCY---CPRGGVILYQDAFTD   265
```
