# Supplementary figures and images for: Seminal vesicle secretory protein 7, PATE4, is not required for sperm function but for copulatory plug formation to ensure fecundity
Source: Biol Reprod. 2018 Nov 18;100(4):1035–45. doi: 10.1093/biolre/ioy247 (PMC6483057; doi:10.1093/biolre/ioy247)

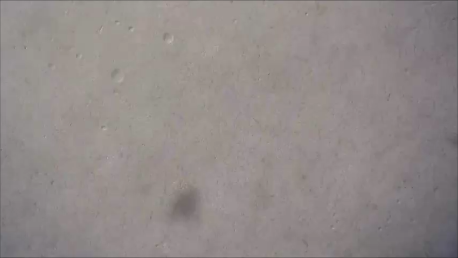

Supplement: Supplemental Files [file ioy247_supplemental_files.zip › Supplemental Movie S1 (still image, Noda et al.).png]

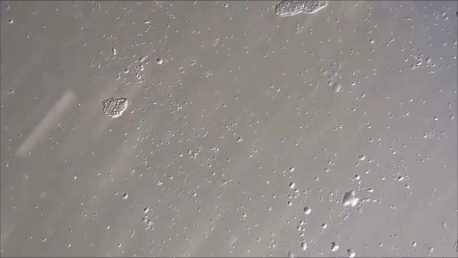

Supplement: Supplemental Files [file ioy247_supplemental_files.zip › Supplemental Movie S2 (still image, Noda et al.).png]

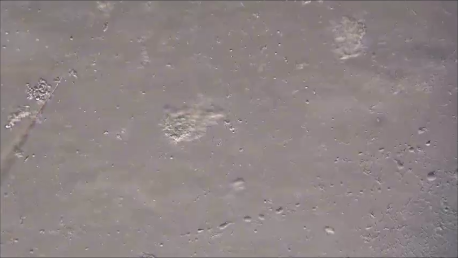

Supplement: Supplemental Files [file ioy247_supplemental_files.zip › Supplemental Movie S3 (still image, Noda et al.).png]

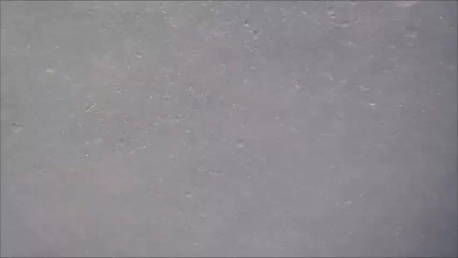

Supplement: Supplemental Files [file ioy247_supplemental_files.zip › Supplemental Movie S4 (still image, Noda et al.).png]

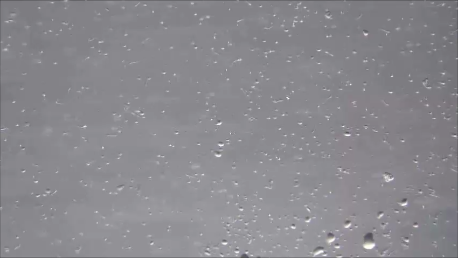

Supplement: Supplemental Files [file ioy247_supplemental_files.zip › Supplemental Movie S5 (still image, Noda et al.).png]
